# Impact of *Agaricus bisporus* Mushroom Consumption on Gut Health Markers in Healthy Adults

**DOI:** 10.3390/nu10101402

**Published:** 2018-10-02

**Authors:** Julie Hess, Qi Wang, Trevor Gould, Joanne Slavin

**Affiliations:** 1 Department of Food Science and Nutrition, University of Minnesota, 1334 Eckles Avenue, St. Paul, MN 55108, USA; jmhess@umn.edu; 2 Clinical and Translational Science Institute, University of Minnesota, 717 Delaware St. SE, Minneapolis, MN 55414, USA; wangx890@umn.edu; 3 Informatics Institute, University of Minnesota, 101 Pleasant St., Minneapolis, MN 55455, USA; goul0109@umn.edu

**Keywords:** mushrooms, gut health, laxation, prebiotic, fiber, microbiota

## Abstract

Eating *Agaricus bisporus* mushrooms may impact gut health, because they contain known prebiotics. This study assessed mushroom consumption compared to meat on gastrointestinal tolerance, short chain fatty acid (SCFA) production, laxation, and fecal microbiota. A randomized open-label crossover study was conducted in healthy adults (*n* = 32) consuming protein-matched amounts of mushrooms or meat twice daily for ten days. Breath hydrogen measures were taken on day one, and gastrointestinal tolerance was evaluated throughout treatments. Fecal sample collection was completed days 6–10, and samples were assessed for bacterial composition, SCFA concentrations, weight, pH, and consistency. There were no differences in breath hydrogen, stool frequency, consistency, fecal pH, or SCFA concentrations between the two diets. The mushroom diet led to greater overall gastrointestinal symptoms than the meat diet on days one and two. The mushroom-rich diet resulted in higher average stool weight (*p* = 0.002) and a different fecal microbiota composition compared to the meat diet, with greater abundance of Bacteroidetes (*p* = 0.0002) and lower abundance of Firmicutes (*p* = 0.0009). The increase in stool weight and presence of undigested mushrooms in stool suggests that mushroom consumption may impact laxation in healthy adults. Additional research is needed to interpret the health implications of fecal microbiota shifts with mushroom feeding.

## 1. Introduction

Dietary fiber and other low and non-digestible carbohydrates are considered important nutrients for human health [1,2,3,4]. Many studies have been conducted on their benefits both when added to the diet as supplements (in isolated forms) [5,6,7] and when provided as part of a food [8,9,10,11]. Some health benefits linked with fiber consumption include a reduced risk of cardiovascular disease [2], enhanced satiety, reduced postprandial blood glucose, and improved laxation [12]. Recent research suggests that consumption of fiber may also benefit the gut microbiota, especially since some fibers also function as prebiotics [2]. Certain foods like bran cereal, beans and legumes, and some fruits and vegetables are considered good sources of dietary fiber [13], and therefore, are recommended in U.S. Dietary Guidance [1] as foods to eat in order to consume adequate dietary fiber (25 g daily for women, 38 g daily for men) [12]. However, some foods, including mushrooms, that do not qualify for a “good source of fiber” label according to U.S. Food and Drug Administration (FDA) guidance [13], still contain low and non-digestible carbohydrates and may benefit gut health.

The carbohydrate profile of mushrooms, which includes several different types of low-digestible and non-digestible carbohydrates, including chitin, β-glucans, raffinose, oligosaccharides, and resistant starch [14,15,16,17], suggests that they may improve laxation, stimulate short-chain fatty acid (SCFA) production, and impact gut microbial populations. Even common *Agaricus bisporus* mushrooms, or white button mushrooms, have a unique carbohydrate profile that includes low-digestible carbohydrates, such as resistant starch, β-glucans, and mannitol, known to have gastrointestinal effects [4,15,16,18,19].

These low-digestible carbohydrates have been evaluated for their effects on gastrointestinal health or function when provided in isolated forms. Resistant starch isolated from mushrooms has not been evaluated for its impact on gut health. Some [20], but not all [21], studies on resistant starch from other sources show that it has a beneficial impact on laxation markers. This effect was primarily seen with doses ≥25 g per day. Similarly, the health impact of fungal β-glucans isolated specifically from white button mushrooms has not been evaluated to our knowledge. However, isolated fungal β-glucans from Shiitake mushrooms (*Lentinus edodes*) and Oyster mushrooms (*Pleurotus ostreatus*), both from the same taxonomic order (Agaricales) as *Agaricus bisporus*, have elicited a beneficial impact on intestinal health in studies with animal models [22]. Relatively few studies have been conducted on the gastrointestinal effects of mannitol ingestion. A 2009 review describes that mannitol is well tolerated in doses up to 20 g daily but may lead to diarrheal stools at amounts higher than 40 g [4]. In fact, the FDA requires a warning label (“Excess consumption may have a laxative effect”) on foods that could reasonably provide 20 g or more of mannitol in one day [23]. Mannitol has also been referred to as a prebiotic, or a “substrate selectively utilized by host microorganisms to confer a health benefit” [24], in an animal model [25].

These components (resistant starch, β-glucans, and mannitol) have not been evaluated for their impact on human gut health when consumed in white button mushrooms (“mushrooms”). The gastrointestinal impact of eating these mushrooms has so far only been evaluated in animal studies. Results from animal studies suggest that some carbohydrates in mushrooms function as prebiotics in a mouse model as well as in turkey poults and broiler chickens. Adding 1% freeze-dried and ground white button mushrooms to the purified diet of C57BL/6 mice resulted in increased gut bacterial diversity, including increases in Bacteroidetes and decreases in Firmicutes compared with control-fed mice [26]. While not model animals, turkey poults fed *A. bisporus* mushrooms added at 0, 10, or 20 g/kg feed for 70 days increased ileal *Lactobacilli* spp. counts (*p* = 0.000) in both the 10 g/kg and 20 g/kg groups compared to the control group [27]. Ileal *E. coli* populations were also significantly lower (*p* = 0.043) in the 20 g/kg groups compared to the other two groups. In addition, cecal *Lactobacilli* spp. (*p* ≤ 0.05) was higher in both mushroom-supplemented groups and *Bifidobacterium* spp. was higher in the 20 g/kg group (*p* = 0.045). A similar experiment [28] conducted by the same research team found that adding 0, 10, or 20 g/kg of ground, dried mushrooms to the feed of broiler chickens for 42 days increased ileal *Lactobacilli* spp. populations (*p* = 0.005) in the 20 g/kg group. The mushroom diet also slightly increased cecal *Lactobacilli* spp. and *Bifidobacteria* spp. (*p* = 0.005) in both supplemented groups [28]. Two additional studies conducted in broiler chickens found that adding ground, dried mushrooms in amounts ranging from 10 g/kg of feed to 30 g/kg of feed decreased *E. coli* levels [29,30] compared to control diets, and in one study, also significantly increased *Lactobacilli* spp. [30]. The authors of each of these studies concluded that including mushrooms in the diet of these animals beneficially influenced gastrointestinal health and intestinal microbial communities.

While this research in animals is promising, and the impact of mushrooms on gut health in humans has been the subject of scientific speculation for several years [31,32,33,34,35,36], it has not been formally evaluated in a clinical trial prior to this study to our knowledge. The objective of this study was to assess the impact of 10 days of mushroom consumption compared to meat consumption on gut health markers and the fecal microbiota of healthy adults. Mushrooms were compared to meat in this study, because, as addressed in a previous manuscript [14], this study was also designed to build on research on the satiety impact of mushrooms. In other satiety research on this topic, mushrooms have been compared to meat and specifically to beef. In addition to evaluating changes in the fecal microbiota, this study also assessed other endpoints that have been tied to bacterial fermentation of prebiotics, including short chain fatty acid (SCFA) concentrations [2] and laxation markers [2,5] such as stool weight, pH, and consistency. This study also collected subjective measures of gastrointestinal (GI) tolerance as well as breath hydrogen and breath methane measurements, which serve as markers of colonic fermentation by gas-producing bacteria. To our knowledge, none of these outcomes have been evaluated in a human population with mushroom feeding.

Given the health effects observed with isolated forms of the carbohydrates found in mushrooms [22,25,37], we hypothesized that mushroom feeding would result in greater abundance of beneficial bacteria (including *Bifidobacteria* and *Lactobacilli*) in the fecal microbiota as well as a higher concentration of SCFA relative to a meat control. We also hypothesized that the mushroom treatment would be well-tolerated by participants and result in improved laxation markers, including greater fecal bulk and a higher rate of “normal” stool consistency, compared to the meat treatment.

## 2. Materials and Methods

### 2.1. Subjects

Participants were recruited by flyers on the University of Minnesota’s St. Paul campus and asked to complete an online screening survey (Qualtrics, Provo, UT, USA). Healthy men and women between the ages of 18 and 65 with a body mass index between 18.5 and 30 kg/m^2^ were eligible. All participants also had to be regular breakfast and lunch consumers (≥4 times per week) willing to consume both meat and mushrooms. Individuals were not eligible to participate if they had a serious preexisting health condition (diabetes, kidney/liver disease, cancer, and eating disorder) and/or were taking medication for blood sugar, cholesterol, blood pressure, or weight loss. Individuals taking laxatives or anti-diarrheal medications or individuals who had gained or lost more than 10 pounds in the last three months were also ineligible to participate. Pregnant or lactating females were excluded. Participants could not be regular fiber consumers (had to consume ≤3 servings of fiber-rich foods per day) and could not take supplements besides a multivitamin. Participants could not have been on antibiotics within the last three months and could not have any had any gastrointestinal conditions or surgeries. Additional inclusion and exclusion criteria as well as detailed participant demographics have been published elsewhere [14].

**Figure 1:**
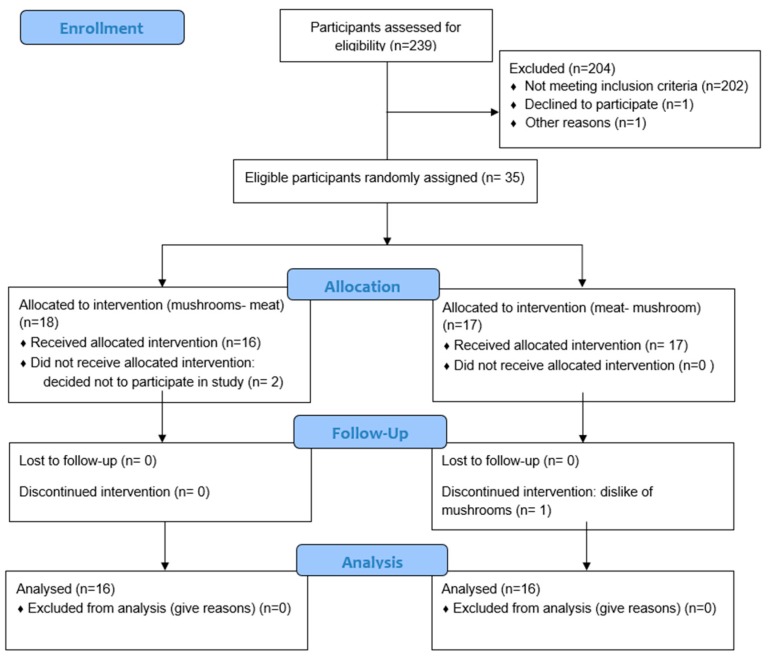
CONSORT flow diagram depicting the flow of participants through the intervention trial. CONSORT, Consolidated Standards of Reporting Trials.

Thirty-five participants completed the informed consent process. Two female participants dropped out of the study before attending any sessions due to scheduling conflicts, and one male participant dropped out of the study halfway through due to dislike of mushrooms. Thirty-two participants (17 females, 15 males) completed the entire study (Figure 1). The University of Minnesota Institutional Review Board Human Subjects Committee reviewed and approved all methods for human participants, and all participants provided written informed consent. This trial was registered at clinicaltrials.gov as NCT03352050.

### 2.2. Experimental Design and Treatments

This study used a randomized, open-label crossover design to assess the difference on gut health outcomes of protein-matched amounts of mushrooms and meat. The amounts of mushrooms (sliced, raw; Giorgio) and meat (93% lean ground beef; Market Pantry) were matched for protein because this experiment was also part of a satiety study conducted in our laboratory [14]. Each serving of mushrooms also contained approximately 6 g of candidate prebiotics (Table 1), exceeding the 3 g per day identified as the minimum oral dose required to elicit an effect by the International Scientific Association for Probiotics and Prebiotics [24].

Participants completed one in-person visit at the beginning of each experimental treatment (mushrooms and meat). At in-person visits, participants were given breakfast sandwiches containing sliced, roasted mushrooms (226 g) or meat (28 g) [14]. Gastrointestinal tolerance and breath hydrogen were assessed at baseline and at regular intervals throughout each 3.5 h in-person visit. Upon leaving the in-person study visit, participants were given a serving of mushrooms or meat to consume at dinner that night as well as at breakfast and at dinner for the following nine days (Table 2). Participants performed a five-day total fecal collection the last five days (days 6 to 10) of each treatment period and had a minimum ten-day washout period between treatments.

### 2.3. Gastrointestinal Tolerance

Participants completed GI tolerance questionnaires on three days of each ten-day feeding treatment. At each in-person visit, participants completed questionnaires at baseline, and at 60, 120, and 180 min after baseline as well as 12 h after baseline (8:00 p.m.). Participants were asked to complete GI tolerance questionnaires at the same times (8:00 a.m., 9:00 a.m., 10:00 a.m., 11:00 a.m., and 8:00 p.m.) on days two and ten of each treatment period.

These questionnaires required participants to rate the severity of specific gastrointestinal symptoms they experienced. GI tolerance of the mushroom and meat treatments were measured with seven different symptoms (gas/bloating, nausea, flatulence, diarrhea, constipation, GI cramping, GI rumbling). Participants ranked symptom severity using a 4-point Likert scale (“none”, “mild”, “moderate”, and “severe”). While no GI tolerance scale has been validated in a healthy population to our knowledge, this scale has been used in previous studies conducted in our lab to assess tolerance [8,38,39].

### 2.4. Colonic Fermentation

Breath samples were collected at baseline and at 60, 90, and 180 min after baseline. Subjects were asked to fill a sample collection bag (750 mL) with air. All breath samples were analyzed using the same instrument, a BreathTracker (QuinTron Instrument Company, Milwaukee, WI, USA). For analysis, 20 mL of a breath sample was injected into the BreathTracker. Breath hydrogen and breath methane samples were all evaluated twice for each sample for greater accuracy. The two measurements were averaged before computing final results.

### 2.5. Fecal Collection

Participants were given specimen collectors (JDC Supplies Commode Specimen Collector Pans 1200 mL DYND36500) and anaerobic pouches (AnaeroPack from Mitsubishi Gas Chemical America, New York, NY, USA) as well as coolers and ice packs to collect samples. Participants were instructed to bring their samples, on ice in insulated coolers, to the lab within 2 h of defecation.

Samples were processed within one hour of their arrival in the lab. Samples were weighed and assessed for Bristol scale [40] by visual comparison with Bristol scale pictures and written descriptions. Fecal samples were then divided into aliquots for different experiments. Samples for branched-chain fatty acid (BCFA) and SCFA determination and microbial analysis were immediately frozen at −80 °C until analysis.

### 2.6. Fecal pH

For fecal pH, a 10 g aliquot of fresh fecal sample was diluted 1:10 (*w*/*w*) with phosphate buffered solution (PBS). The fecal/PBS mixture was homogenized in a stomacher for 2 min, and the homogenized sample then used for pH measurement using a calibrated pH probe.

### 2.7. BCFA and SCFA Analysis

Fecal samples were analyzed for BCFA and SCFA content using the extraction and derivatization procedures described by Han et al. [41]. Briefly, 1 g of fecal sample was combined with 10 mL 50% aqueous acetonitrile, and the mixture homogenized with a vortex. Then samples were centrifuged at 4000 *g* at 10 °C for 10 min. The clarified extract was then diluted 1:10 with 50% aqueous acetonitrile and 9 µM of internal standard added. Samples were stored at −80 °C until analysis. Before analysis, 20 µL each of 3-Nitrophenylhydrazine hydrochloride solution and *N*-(3-Dimethylaminopropyl)-*N*′-ethylcarbodiimide hydrochloride solution were added to 40 µL of the extracted sample and the mixture incubated at 40 °C for 30 min.

Samples (10 µL) for LC-MS/MS Selective Reaction Monitoring (SRM) Analysis of SCFA and BCFA were subjected to separation using a Shimazu UFLCXR system coupled to an analytical Waters Aquity BEHc18, 1.7 µm, 2.1 × 50 mm column at 50 °C connected to the Applied Biosystem 5500 iontrap fitted with a turbo V electrospray source run in negative mode with declustering potential and collision energies (Supplemental Table S1). The samples were subjected to a linear gradient of A: 15% acetonitrile 0.55 formic acid B: 55% acetonitrile 0.1% formic acid for 12 min at a column flow rate of 400 µL/min. The column was cleared with 95% acetonitrile for 2 min and then equilibrated to buffer A for 3 min. Transitions monitored as in Table S1 were established using the instrument’s compound optimization mode with direct injection for each compound. The data was analyzed using MultiQuant™ (ABI Sciex Framingham, MA, USA) providing the peak area. A standard curve was constructed using from picomole to nanomole in 10 µL. Samples were run in duplicate and concentrations determined from the standard curve. SCFA and BCFA concentration values for each participant were averaged across all samples submitted during each treatment.

### 2.8. Microbial Analysis

#### 2.8.1. DNA Extractions

Fecal bacteria DNA were extracted using the MO BIO PowerSoil DNA Isolation Kit (MO BIO Laboratories, Inc., Carlsbad, CA, USA) following the provided operating instructions.

#### 2.8.2. Amplification, Quantification, and Sequencing

After extraction, the V1–V3 region of the 16S rRNA was amplified using a dual-indexing approach described in an Illumina technical note [42]. PCR products were quantified using a PicoGreen dsDNA assay kit (Life Technologies, Carlsbad, CA, USA). A detailed description of methods used has been previously published [43]. Samples were sequenced using Illumina HiSeq 2500 Rapid Mode at the University of Minnesota Genomics Center.

#### 2.8.3. Sequence Processing and Analysis

Generated sequence data was processed for sequence quality and analyzed using hybrid-denovo [44] and QIIME [45] using default parameters. Fastq sequence data was processed by the Mayo Clinic Bioinformatic Core using their hybrid-denovo workflow [44].

### 2.9. Statistical Analysis

Gut health and fermentation endpoints (GI tolerance, colonic fermentation, laxation markers, SCFA and BCFA concentrations): Our sample size was selected to give us at least 80% power to detect a significant mean difference of 0.7 SD in markers of gut health between the two diets. Paired t-tests were conducted to compare the means between the two diets. Analyses were performed using the Statistical Analysis System software (SAS, version 9.3, 2011; SAS Institute, Cary, NC, USA). *p*-Values < 0.05 were considered statistically significant.

Percent abundance of bacterial sequences in fecal microbiota were analyzed using linear mixed models, and adjusted for age, sex and BMI, in which the outcome was log-transformed due to the skewed distribution. After controlling for false discovery rate, *p*-values < 0.004 should be considered as statistically significant for fecal microbiota data.

### 2.10. Compliance

Participants were provided tracking checklists to record which days they ate and did not eat provided study foods. Participants were also asked to complete 24-h food diaries for days 1 (in-person visit), 2, and 10.

## 3. Results

### 3.1. Breath Hydrogen

There were no differences in breath hydrogen or in breath methane between the two treatments as assessed with baseline-corrected AUC measurements (Table 3).

### 3.2. Gastrointestinal Tolerance

On Day 1, participants reported significantly more total GI symptoms during the mushroom treatment in comparison to the meat treatment (Table 4). Significant individual GI symptoms included gas and flatulence only. On Day 2, participants also reported significantly more total GI symptoms, gas, and flatulence during the mushroom treatment (Table 5). On the last day of eating each study diet (Day 10, Table 6), there were no significant differences between the two diets with any of the GI symptoms.

### 3.3. Laxation Markers

Participant stool weights were significantly higher on the mushroom treatment than the meat treatment (*p* = 0.002) (Table 7). However, there were no significant differences between the two diets in terms stool frequency, pH, or consistency (Table 7).

### 3.4. SCFA and BCFA Concentrations

There were also no significant differences between acetate, propionate, butyrate, isobutyrate, or valerate concentrations between the two treatments (Table 8). There was a significant difference with isovalerate concentration (*p* = 0.02), which was higher in the meat control diet.

### 3.5. 16S Sequencing

Sequencing of 261 fecal samples from 32 participants using Illumina HiSeq generated sequences representing 11 phyla, 68 families, and 69 genera of bacteria. Taxa with greater than 0.05% overall abundance in the mushroom and meat diets are summarized in Table 9.

The majority of the sequences were comprised of a few taxa. During both diets, the Bacteroidetes and Firmicutes phyla were the most abundant, consisting of approximately 87% of total sequence reads. With the exception of phyla that could not be classified, Proteobacteria and Verrucomicrobia were the third and fourth most abundant phyla, accounting for approximately 3% and 2% of sequence reads, respectively. Each of the remaining six phyla accounted for less than 2% of sequence reads.

At the genus level, with the exception of genera sequences that could not be classified, *Bacteroides* was the most abundant genus across samples from both diets, accounting for 24% and 19% of sequences in the mushroom and meat diets, respectively. The genera *Bacteroides*, *Parabacteroides*, *Coprococcus*, *Sutterella*, and *Anaerostipes* were found in significantly greater amounts during the mushroom diet than during the meat diet. *Dorea* and *02d06*, members of the Clostridia class, were found in greater abundance during the meat diet.

### 3.6. Compliance

For the mushroom diet, 20 participants turned in fully completed checklists. Five participants turned in mostly complete checklists with five or fewer missed servings. Seven participants turned in blank checklists or were missing checklists altogether. For the meat diet, 22 participants turned in fully complete checklists, four turned in partially completed checklists, and six participants had missing or blank checklists. All participants brought in at least one fecal sample during each collection period. In addition, approximately one-third (31.25%) of participants had visible pieces of mushroom in stool samples at least once during the mushroom treatment, indicating some level of compliance with treatment protocol.

## 4. Discussion

The objective of this study was to assess the effect of mushroom consumption compared to a meat control on markers of gut health. While the mushroom treatment contained low-digestible carbohydrates and the meat treatment did not, there were only a few key differences in impact on gut health markers between the two treatments.

Both treatments were generally well-tolerated by participants in this study, and no adverse symptoms were reported with either treatment. Total GI tolerance scores as well as gas and flatulence ratings were significantly higher on the first two days of the mushroom treatment. The mushroom treatment provided only an additional 6 g of fiber to our participants’ diets. However, since study participants were low fiber consumers, even the 6 g addition may have contributed to increased GI symptoms. An intervention trial with legumes found that adding 4–7 g of fiber daily from beans initially caused an increase in perceived flatulence, but GI tolerance scores returned to normal after a few weeks of daily bean consumption [46]. A similar effect may have occurred in our study, as there were no significant differences in GI tolerance ratings on day 10. Participants may have adjusted to eating a large quantity of mushrooms after ten days, or, as reported in our previous publication on this study [14], participants may have decreased fiber from other sources over the ten-day period, also decreasing their symptoms. Since participants completed subjective questionnaires at the same time on each day of the intervention, GI symptoms could also have been caused by dietary or lifestyle factors besides the study treatments.

Breath hydrogen and breath methane values did not differ significantly between the two treatments. While we did not expect a difference in breath methane, since elevated breath methane values primarily indicate whether an individual is a “methane producer” with colonic methanogenic colonies [47], the lack of difference in breath hydrogen measures may be due to a limitation in our methods. The final breath hydrogen measurement in our study was taken 180 min after treatment intake, which was likely not sufficient time for transit of treatment foods to the colon [48].

Some other measures of colonic fermentation, including fecal pH and SCFA concentrations, also did not differ significantly between the two treatments. Colonic fermentation, which leads to the formation of acids, including SCFA, can acidify the stool [2]. The lack of significant difference in either fecal pH values or SCFA concentrations between the two treatments suggests that little colonic fermentation occurred and the SCFA generated may have been quickly absorbed instead of excreted.

The SCFA findings do not support our hypothesis. Unlike meat, mushrooms contain resistant starch, which tends to increase amounts of SCFA when it reaches colonic bacteria, according to previous research [37]. However, the presence of undigested mushrooms in some participants’ stool indicates that the mushroom treatment was only partially broken down by digestive processes that occur after chewing. Some of the resistant starch or other components provided by the mushroom treatment may not have been available to colonic bacteria. The assays used to determine the low-digestible carbohydrate content of the mushroom treatment (described in Table 1 footnotes) were conducted with roasted mushrooms that had been ground into a well-blended and homogenous sample, which is not reflective of how food is digested in vivo [49,50]. The availability of low-digestible carbohydrates in mushrooms may depend on how thoroughly the mushrooms were chewed by participants. The cell wall of mushrooms is made up of insoluble β-glucans [51] and chitin [52], and humans do not have digestive enzymes to break down those components. It may be that the degree to which the mushrooms were chewed by participants before being swallowed affected the extent to which these components were fermented in the gut.

With BCFA, there was a significant difference between the treatments with isovalerate concentration, which was higher during the meat diet (*p* = 0.02). While SCFA production appears to be largely beneficial and indicates the production of energy for colonocytes, among other benefits [2,4,53,54], BCFA production indicates proteolysis occurring in the large intestine and is presumed to be detrimental to health [53,54]. BCFA are formed in the gut when branched chain amino acids (valine, leucine, and isoleucine) are metabolized and fermented [55,56]. Isovalerate specifically is formed by the breakdown of leucine. While the amount of meat provided in our experimental treatment was smaller than a typical serving (2 oz/day) and participant diet records indicated no significant differences in protein intake during the two interventions [14], beef contains more leucine (1.267 g/100 g) [17] than mushrooms (0.120 g/100 g) [17], which may be responsible for the elevated fecal isovalerate concentrations.

While breath hydrogen, fecal pH, and SCFA findings do not indicate fermentation occurring during the mushroom diet, the fecal microbiota and increase in fecal bulk suggest that the mushroom treatment may have stimulated some colonic fermentation [2]. Fecal microbiota results from this study resemble the results from model animal studies that involved a mushroom intervention [26]. As in the mouse model study [26], in this study, Bacteroidetes was significantly more abundant during the mushroom treatment. The Firmicutes phyla, including the potentially pathogenic *Clostridia* class of bacteria, was less abundant during the mushroom treatment in the fecal microbiota of participants in this study. Studies in model animals have shown lean mice to have less Bacteroidetes than Firmicutes in their cecal microbiota [57]. Similarly, in human studies involving weight loss interventions, individuals losing weight tend to have an increase in Bacteroidetes and decrease in Firmicutes in their fecal microbiota, suggesting a potential role of these phyla in energy homeostasis [2,58]. The results of this study indicate that frequent mushroom consumption may be a way to increase Bacteroidetes abundance relative to Firmicutes, however, the health implications of alterations in their relative abundance in a healthy adult population are not well understood [2].

*Lactobacilli* and *Bifidobacteria* abundance did not align with our hypothesis that the mushroom treatment would lead to increases in both genera. In this study, *Lactobacilli* and *Bifidobacteria* only accounted for a small portion of the microbes present (approximately 0.2% and 0.01%, respectively) during both treatments, with no significant differences between them. Similarly, low *Lactobacilli* and/or *Bifidobacteria* counts have been observed in other studies with healthy adults [7,59] and could be due to a wide range of factors, including environmental exposures, stress, and even cultural traditions [2,60]. Unfortunately, it is not possible to compare *Lactobacilli* and *Bifidobacteria* abundance in this study with a baseline sample. A significant limitation to the fecal microbiota and gut health data in this study is that no baseline fecal samples were collected, so only comparisons between the mushroom and meat diets are possible.

According to both the FDA and the Institute of Medicine (IOM), improved laxation, or the elimination of fecal waste, is considered a beneficial physiological effect of fiber intake [61,62]. Both groups consider stool frequency, ease of defecation, and in some contexts, fecal weight or fecal bulk as markers of improved laxation [61,62]. In this study, mushroom consumption improved laxation as measured by one of these metrics (fecal weight) but not the others (stool frequency and ease of defecation). Stool consistency (ease of defecation) and frequency did not differ between the two diets. Stool consistency or form measures (Bristol score) estimate whole gut transit time and ease of defecation [40]. While there are no official ‘cut-offs’ associated with health or unhealthy states with the Bristol scale, scores 3 and 4 are sometimes referred to as “normal” stool types because they are not associated with urgency, straining, or incomplete evacuation [63]. The average stool type for participants on both diets was between a 3 and 4 on the Bristol scale (i.e., soft, but formed stools), suggesting that both treatments allowed for “normal” laxation. While stool frequency (e.g., number of stools per day) did not differ between the two treatments, in line with our hypothesis, fecal bulk (stool weight) was significantly higher with the mushroom treatment. Yet, FDA draft guidance from 2016 states that “an increase in fecal weight does not necessarily indicate improved bowel function” [61]. Increased fecal weight may not necessarily indicate improved laxation, but it does indicate the presence of a fiber source “slowly, incompletely, or essentially not fermented in the large intestine” [64], which aligns with our other findings. The laxation outcomes of this study may also be subject to limitations. While participants were asked to bring in all stool samples for each five-day period within two hours and record defecation time, we do not know if all stool samples were submitted within that time frame or if all samples were submitted.

## 5. Conclusions

The results of this study, especially the increase in fecal bulk, the lack of an increase in SCFA production compared to the meat diet, and the presence of undigested mushroom in the stool, suggest that mushrooms may be only partially fermentable by human colonic bacteria and that the “low-digestible carbohydrates” in mushrooms also include some non-digestible carbohydrates. Further research is needed to determine whether chewing or other digestive processes could increase the availability of low-digestible carbohydrates in mushrooms. The mushroom diet did result in a different fecal microbiota than the meat diet, similar to the changes observed in studies with model animals. It also increased fecal bulk, which suggests that colonic bacteria may have been able to utilize some portion of the low-digestible carbohydrates in mushrooms. However, further investigation on the fecal microbiota is needed to assess the potential health implications of these differences.

## Figures and Tables

**Table 1:** Carbohydrate content of roasted *A. bisporus* mushrooms ^1^.

Carbohydrate	Percentage of Roasted *A. bisporus* Mushrooms
Total Dietary Fiber	4.9%
Insoluble Dietary Fiber	3.5%
Soluble Dietary Fiber	1.4%
Beta-glucan	1.76%
Mannitol	2.96%
Resistant starch	<2%

^1^ Amounts determined by Medallion Laboratories 1/4/17 using AOAC 2011.25 for fiber determination, AOAC: 2022.02 for resistant starch, and an internal method for sugar alcohols determination using the same brand of *A. bisporus* mushrooms (roasted) utilized in the study.

**Table 2:** Nutrition composition of each serving of mushrooms and meat provided for ten-day food intake assessment.

Treatment Type	Kcal	Total Fat (g)	Carbohydrates (g)	Fiber (g)	Protein (g)
Mushroom (226 g)	50	0	6	3	6
Beef (28 g)	43	2	0	0	6

**Table 3:** Breath hydrogen and breath methane results.

	Mushroom (*n* = 32)	Meat (*n* = 32)	*p*-Value ^b^
Breath Hydrogen ^a^	−12.37 ± 30.83	−12.68 ± 19.77	0.96
Breath Methane ^a^	−0.20 ± 10.78	−2.03 ± 7.76	0.41

^a^ Mean baseline-adjusted area under the curve ± SD. ^b^ *p*-values < 0.05 considered significant.

**Table 4:** Gastrointestinal tolerance measures day 1.

GI Symptom	Mushrooms (*n* = 32)	Meat (*n* = 32)	*p*-Value ^b^
GI Tolerance ^a^	7.88 ± 7.99	4.09 ± 10.63	0.0491
Nausea ^a^	0.06 ± 1.27	−0.19 ± 1.01	0.35
Flatulence ^a^	2.95 ± 2.95	0.67 ± 2.63	0.0002
Diarrhea ^a^	−0.14 ± 0.99	0.19 ± 1.11	0.22
Constipation ^a^	0.36 ± 1.43	0.66 ± 2.11	0.52
Gastrointestinal rumbling ^a^	0.94 ± 2.46	0.61 ± 3.60	0.64
Gastrointestinal cramping ^a^	0.75 ± 2.01	0.80 ± 2.47	0.92
Gas ^a^	2.95 ± 4.05	1.36 ± 3.07	0.0452

^a^ Mean baseline-adjusted area under the curve ± SEM. ^b^ *p*-values < 0.05 considered significant.

**Table 5:** Gastrointestinal tolerance measures day 2.

GI Symptom	Mushrooms (*n* = 32)	Meat (*n* = 32)	*p*-Value ^b^
GI Tolerance ^a^	7.75 ± 8.47	2.95 ± 5.77	0.0042
Nausea ^a^	0.28 ± 2.14	0.16 ± 0.92	0.75
Flatulence ^a^	2.38 ± 2.84	0.61 ± 2.54	0.0156
Diarrhea ^a^	0.58 ± 1.51	0.36 ± 1.00	0.50
Constipation ^a^	0.11 ± 0.89	0.66 ± 1.63	0.11
Gastrointestinal rumbling ^a^	1.50 ± 3.27	0.47 ± 2.46	0.16
Gastrointestinal cramping ^a^	0.86 ± 2.42	0.42 ± 1.85	0.35
Gas	2.05 ± 3.31	0.28 ± 1.55	0.0051

^a^ Mean baseline-adjusted area under the curve ± SEM. ^b^ *p*-values < 0.05 considered significant.

**Table 6:** Gastrointestinal tolerance measures day 10.

GI Symptom	Mushrooms (*n* = 32)	Meat (*n* = 32)	*p*-Value ^b^
GI Tolerance ^a^	5.59 ± 8.83	3.86 ± 7.67	0.36
Nausea ^a^	0.20 ± 0.91	−0.06 ± 0.96	0.094
Flatulence ^a^	1.11 ± 2.46	0.55 ± 2.38	0.38
Diarrhea ^a^	0.45 ± 1.52	0.22 ± 1.57	0.56
Constipation ^a^	0.75 ± 2.23	0.52 ± 1.73	0.65
Gastrointestinal rumbling ^a^	1.09 ± 3.15	1.31 ± 3.88	0.70
Gastrointestinal cramping ^a^	0.56 ± 2.43	0.27 ± 1.66	0.55
Gas	1.42 ± 2.56	1.06 ± 2.73	0.59

^a^ Mean baseline-adjusted area under the curve ± SEM. ^b^ *p*-values < 0.05 considered significant.

**Table 7:** Laxation Markers for fecal samples collected days 6 through 10.

Laxation Measure	Mushroom (*n* = 32)	Meat (*n* = 32)	*p*-Value ^d^
Fecal wet weight (g/stool) ^a^	122.42 ± 58.74	94.62 ± 56.58	0.002
Stool consistency ^a,b^	3.12 ± 0.89	3.35 ± 0.79	0.11
Fecal pH ^a^	6.86 ± 0.21	6.87 ± 0.16	0.77
Stool frequency ^a,c^	4.25 ± 1.30	4.13 ± 1.26	0.35

^a^ Data presented are mean values ± standard deviation; ^b^ Stool consistency rated on Bristol stool scale, where 1 = separate hard lumps and 7 = entirely liquid. ^c^ Stool frequency provided as number of samples collected days 6 to 10. ^d^ *p*-values < 0.05 considered significant.

**Table 8:** Fecal short chain fatty acids (SCFA) and branched chain fatty acids (BCFA) produced on mushroom and meat diets.

SCFA and BCFA (µM/mL)	Mushrooms (*n* = 32)	Meat (*n* = 32)	*p*-Value ^b^
Acetate ^a^	3930.35 ± 732.34	3825.01 ± 731.01	0.16
Propionate ^a^	64.72 ± 45.04	64.73 ± 34.07	0.99
Butyrate ^a^	53.81 ± 37.69	58.27 ± 33.43	0.46
Isobutyrate ^a^	28.42 ± 25.68	30.76 ± 25.49	0.52
Valerate ^a^	8.02 ± 5.36	9.78 ± 6.30	0.10
Isovalerate ^a^	5.31 ± 2.86	6.92 ± 3.52	0.02

^a^ Mean values ± SD on a wet matter basis. ^b^ *p*-values < 0.05 considered significant.

**Table 9:** Percent abundance across 5 days of fecal collection for identified abundant bacterial taxa.

Targeted Taxa (Phylum and Genus)	Percent Abundance ^1^		*p*-Value ^2^
	Mushroom Diet	Meat Diet	
Actinobacteria	0.65 ± 0.07	1.16 ± 0.30	0.06
*Collinsella*	0.33 ± 0.05	0.73 ± 0.27	0.20
*Bifidobacterium*	0.01 ± 0	0.01 ± 0	0.59
*Adlercreutzia*	0.08 ± 0.02	0.08 ± 0.01	0.27
*Propionibacterium*	0.01 ± 0	0.01 ± 0	0.20
*Actinomyces*	0.01 ± 0	0.03 ± 0.01	0.08
*Eggerthella*	0.02 ± 0.01	0.03 ± 0.01	0.93
*Slackia*	0.05 ± 0.01	0.08 ± 0.03	0.39
Bacteroidetes	37.79 ± 1.41	31.65 ± 1.99	0.0002 *
*Bacteroides*	23.83 ± 1.68	18.91 ± 1.86	0.0001 *
*Parabacteroides*	3.42 ± 0.37	2.20 ± 0.24	0.001 *
*Prevotella*	4.87 ± 1.21	4.56 ± 1.21	0.90
*Odoribacter*	0.26 ± 0.04	0.31 ± 0.08	0.78
*Porphyromonas*	0 ± 0	0.03 ± 0.02	0.02
*Paraprevotella*	0.22 ± 0.12	0.13 ± 0.08	0.65
*Butyricimonas*	0.10 ± 0.03	0.09 ± 0.02	0.59
Firmicutes	49.81 ± 1.54	54.84 ± 1.88	0.0009 *
*Blautia*	5.00 ± 0.58	6.30 ± 0.72	0.04
*Faecalibacterium*	3.77 ± 0.36	3.63 ± 0.29	0.93
*Ruminococcus*	6.20 ± 0.57	7.21 ± 0.79	0.29
*Coprococcus*	5.53 ± 0.35	4.49 ± 0.30	0.0002 *
*Oscillospira*	2.63 ± 0.29	3.32 ± 0.46	0.10
*Lachnospira*	0.93 ± 0.15	0.84 ± 0.16	0.30
*Phascolarctobacterium*	1.77 ± 0.32	1.35 ± 0.24	0.41
*Dorea*	0.99 ± 0.13	1.45 ± 0.19	0.002 *
*Streptococcus*	0.43 ± 0.08	0.44 ± 0.06	0.61
*Dialister*	0.92 ± 0.24	1.30 ± 0.38	0.51
*Clostridium*	0.69 ± 0.12	0.75 ± 0.09	0.05
*Eubacterium*	0.85 ± 0.24	1.07 ± 0.28	0.10
*Veillonella*	0.08 ± 0.03	0.25 ± 0.20	0.38
*Catenibacterium*	0.54 ± 0.28	0.59 ± 0.29	0.58
*Roseburia*	0.93 ± 0.10	1.03 ± 0.14	0.54
*Turicibacter*	0.12 ± 0.03	0.26 ± 0.08	0.02
*SMB53*	0.70 ±0.14	1.28 ± 0.21	0.004
*Anaerostipes*	0.50 ± 0.09	0.18 ± 0.03	0.001 *
*Lachnobacterium*	0.12 ± 0.03	0.15 ± 0.06	0.86
*Lactobacillus*	0.21 ± 0.15	0.25 ± 0.16	0.76
*Megamonas*	0.17 ± 0.16	0.08 ± 0.06	0.23
*Megasphaera*	0.10 ± 0.08	0.31 ± 0.14	0.03
*02d06*	0.25 ± 0.09	0.55 ± 0.15	0.006 *
*Acidaminococcus*	0.08 ± 0.05	0.15 ± 0.08	0.04
*Anaerotruncus*	0.02 ± 0.01	0.03 ± 0.01	0.09
*Bulleidia*	0.01 ± 0.01	0.02 ±0.01	0.53
*cc115*	0.02 ± 0.01	0.03 ± 0.02	0.37
*Coprobacillus*	0.24 ± 0.06	0.26 ± 0.06	0.59
*Holdemania*	0.05 ± 0.01	0.06 ± 0.01	0.86
*Lactococcus*	0.02 ± 0.01	0.04 ± 0.02	0.93
*Mitsuokella*	0.01 ± 0.01	0.04 ± 0.02	0.05
*Mogibacterium*	0.02 ± 0.01	0.02 ± 0.01	0.10
*rc4-4*	0.02 ± 0.01	0.02 ± 0.01	0.08
*Peptococcus*	0.01 ± 0.01	0.01 ± 0.01	0.18
*Dehalobacterium*	0.01 ± 0	0.01 ± 0	0.35
*Succiniclasticum*	0.05 ± 0.04	0.04 ± 0.04	0.31
Proteobacteria	3.08 ± 0.41	2.65 ± 0.49	0.13
*Sutterella*	1.18 ± 0.20	0.76 ± 0.15	0.0006 *
*Bilophilia*	0.20 ± 0.06	0.11 ± 0.03	0.02
*Haemophilus*	0.32 ± 0.09	0.47 ± 0.14	0.34
*Oxalobacter*	0.04 ± 0.01	0.02 ± 0.01	0.13
*Desulfovibrio*	0.16 ± 0.05	0.13 ± 0.05	0.61
*Succinivibrio*	0.21 ± 0.21	0.10 ± 0.10	0.77
Verrucomicrobia	1.71 ± 0.41	1.96 ± 0.47	0.63
*Akkermansia*	1.68 ± 0.41	1.97 ± 0.47	0.53
Fusobacteria	0.19 ± 0.16	0.02 ± 0.01	0.26
*Fusobacterium*	0.19 ± 0.16	0.02 ± 0.01	0.30
Unclassified	4.92 ± 0.48	6.15 ± 0.85	0.03

^1^ Results presented as Mean values ± SE. ^2^ *p*-values < 0.004, designated with * indicate significant difference.

## Supplementary Materials

The following are available online at http://www.mdpi.com/2072-6643/10/10/1402/s1, Table S1: Declustering potential and collision.
