# Supplementary material for: Impact of Agaricus bisporus Mushroom Consumption on Gut Health Markers in Healthy Adults
Source: Nutrients. 2018 Oct 2;10(10):1402. doi: 10.3390/nu10101402 (PMC6213353; doi:10.3390/nu10101402)
Supplement: Supplementary file 1 [file nutrients-10-01402-s001.docx]

**Table S1. Declustering potential and collision**

| **Fatty Acids** | **Q1 M/Z** | **Q2 M/Z** | **Dwell Time** | **Decluster Potential** | **Collision Energy** | **Retention Time** |
| --- | --- | --- | --- | --- | --- | --- |
| Acetate | 194 | 137.1 | 50 | -40 | -20 | 2.8 |
| Propionate | 208 | 165.1 | 50 | -50 | -20 | 4.9 |
| Isobutyrate | 222 | 137.1 | 50 | -65 | -20 | 8.0 |
| 4-Methylvalerate | 250 | 137.1 | 50 | -65 | -20 | 12.2 |
| Valerate | 236.104 | 137.1 | 50 | -65 | -20 | 10.0 |
| Butyrate | 222.08 | 137.1 | 50 | -65 | -20 | 8.3 |
| 2-Ethylbutyric acid | 250.1 | 137.1 | 50 | -65 | -20 | 12.5 |
| Isovalerate | 236.103 | 137.1 | 50 | -65 | -20 | 10.0 |
